# Differential effects of HIF-α isoforms on apoptosis in renal carcinoma cell lines

**DOI:** 10.1186/s12935-015-0175-3

**Published:** 2015-02-22

**Authors:** Alana Doonachar, Michael D Gallo, Donald Doukas, Rajiv Pasricha, Igor Lantsberg, Alan R Schoenfeld

**Affiliations:** Department of Biology, Adelphi University, One South Avenue, P.O. Box 701, Garden City, NY 11530-0701 USA

**Keywords:** Apoptosis, Glucose starvation, HIF-alpha, Serum starvation, UV-irradiation, VHL

## Abstract

**Background:**

Germline mutations in the von Hippel-Lindau (VHL) tumor suppressor gene predispose individuals to clear cell renal carcinomas, hemangioblastomas, and pheochromocytomas. The VHL gene product forms an ubiquitin E3 ligase complex, with regulation of hypoxia-inducible factor alpha (HIF-α) as its best known function. Lack of VHL expression has been shown previously to sensitize renal cells to apoptosis caused by certain cellular stresses. In this report, the role of HIF-α in apoptosis was investigated using two parent VHL-null renal carcinoma cell lines.

**Methods:**

786-O and RCC10 renal carcinoma cell lines with manipulated levels of VHL, HIF-1α, or HIF-2α were subjected to cellular stresses and analyzed by western blotting for the abundance of apoptotic markers.

**Results:**

Cell lines expressing mutant VHL proteins that were unable to regulate HIF-α had increased levels of apoptosis when irradiated with ultraviolet (UV) light. The influences of the two major isoforms of HIF-α, HIF-1α and HIF-2α, on apoptosis, were compared by creating cell lines in which levels of each isoform were modulated via short hairpin RNA interference. In UV-irradiated cells, HIF-2α expression was determined to promote apoptosis, whereas HIF-1α was anti-apoptotic. In cells deprived of either glucose or serum, HIF-1α expression was generally anti-apoptotic, while HIF-2α expression was observed to either promote apoptosis or have less of an influence on apoptosis, depending on the cell line used.

**Conclusions:**

HIF-1α and HIF-2α exerted distinct effects in each of the conditions tested, with expression of HIF-1α largely blocking apoptosis and HIF-2α generally promoting apoptosis. These results reinforce that HIF-1α and HIF-2α have distinct biological roles and that their relative expression levels may influence some therapeutic interventions that are dependent on apoptosis.

## Background

Inactivation of the von Hippel-Lindau (VHL) tumor suppressor gene occurs in VHL disease, an inherited cancer syndrome that predisposes affected individuals to a number of benign and malignant tumors that may affect various organs such as the kidneys, the central nervous system, the retina, and the pancreas [1]. The VHL protein (pVHL) is a part of an ubiquitin E3 ligase complex that targets proteins for proteolysis [2,3]. One important target of pVHL ubiquitination is hypoxia-inducible factor alpha (HIF-α), a subunit of the transcription factor HIF, which up-regulates hypoxia inducible genes [4-6]. Renal cell carcinoma is particularly dependent on loss of functional VHL and the ensuing up-regulation of HIF-α and HIF transcriptional targets [7].

Although loss of VHL can lead to up-regulation of both HIF-1α and HIF-2α isoforms, there are some functional differences that exist between these two isoforms. While there are many common transcriptional targets, it appears that each HIF-α isoform has some distinct genes that it can transactivate [8]. For example, HIF-1α, but not HIF-2α can direct expression of glycolytic genes [9] and HIF-2α, but not HIF-1α can up-regulate expression of cyclin D1, transforming growth factor alpha (TGF-α) and vascular endothelial growth factor VEGF [8,10]. Through opposing functional interactions with the cMyc oncoprotein, HIF-1α has been seen to slow down the cell cycle and tumor growth, whereas HIF-2α has been seen to promote cell proliferation [11]. In renal carcinoma, loss of VHL can lead to sustained up-regulation of both HIF-1α and HIF-2α isoforms in some tumors or only HIF-2α in other tumors, but not solely HIF-1α [12]. Accordingly, it is not surprising that HIF-2α, but not HIF-1α, has been seen as the major driver of renal tumorigenesis [7,13].

Previous studies have shown that VHL expression can protect renal cells against some pro-apoptotic cellular stresses. Reintroduction of pVHL into VHL-deficient cells leads to protection from the cytotoxic effects of serum withdrawal, glucose deprivation, impaired protein processing, chemical hypoxia, and UV radiation [14-17]. It is likely that VHL exerts some of these effects through regulation of HIF-α, although it is presently unclear whether regulation of HIF-1α or HIF-2α is more important for this effect. While some differences in the regulation of pro- and anti-apoptotic genes by HIF-1α and HIF-2α have been described [8,9,18], it has not been fully elucidated whether there are distinct influences of HIF-1α versus HIF-2α on apoptosis due to various stimuli. Here, we carry out a set of experiments to investigate whether expression levels of HIF-1α and HIF-2α have differential effects on apoptosis in renal cells subjected to certain stresses.

## Results

### Apoptosis in UV-treated 786-O cells with mutant VHL proteins correlates to HIF-2α levels

**Figure 1:**
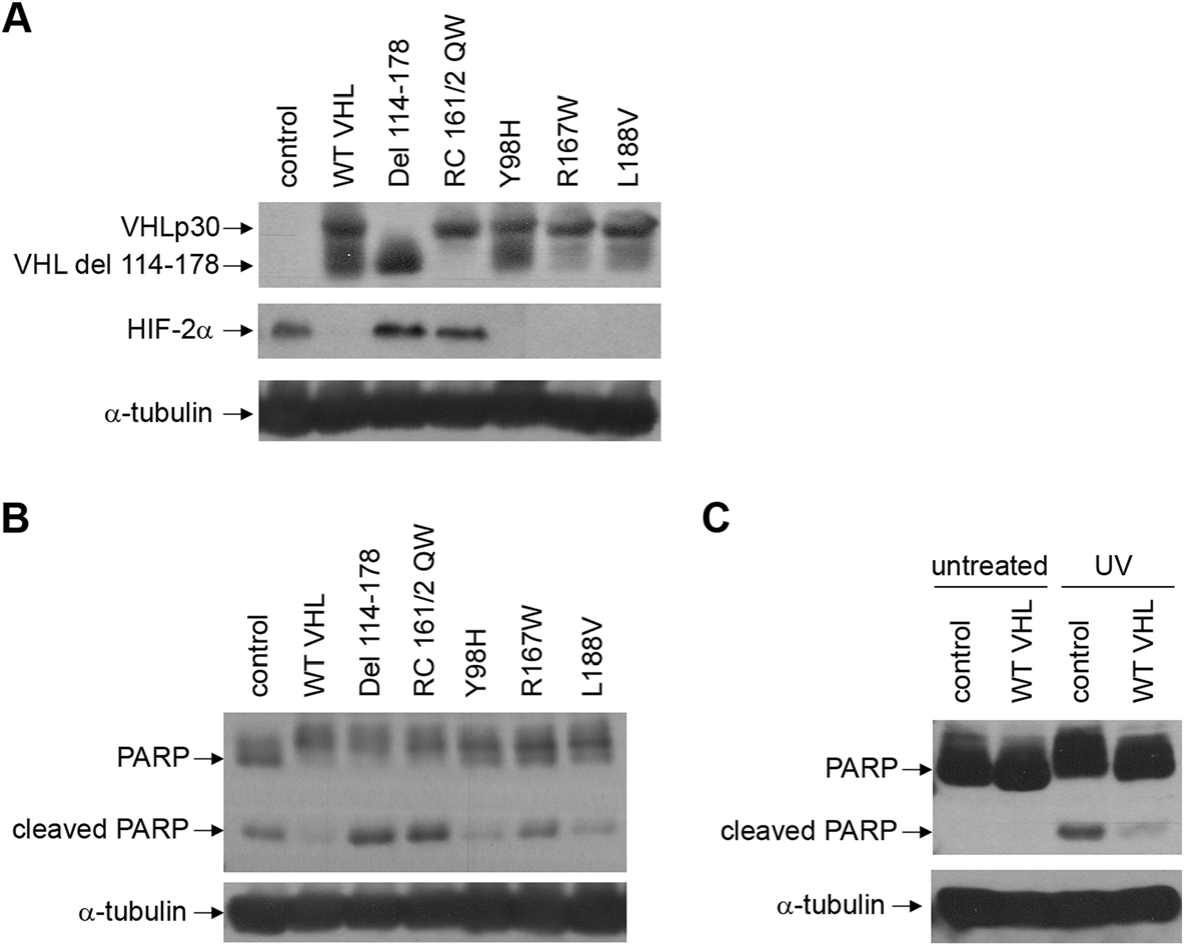
**Apoptosis in UV-treated 786-O cells with mutant VHL proteins correlates to HIF-2α levels. (A)** 786-O cells stably expressing either an empty vector construct (control), wild-type VHLp30 (WT VHL), or various mutant VHLp30 proteins (as indicated) were grown to confluence and lysed. Cell lysates were equally loaded and separated by SDS-PAGE. Western blots were performed for VHL, HIF-2α, and α-tubulin. VHL del 114-178 is a deletion of amino acid residues 114-178, whereas the rest of the VHL mutants are amino acid substitutions. **(B)** The 786-O cell lines in **(A)** were grown to confluence and treated with ultraviolet (UV) light and lysed one day later. Cell lysates were equally loaded and separated by SDS-PAGE. Western blots were performed for poly (ADP-ribose) polymerase (PARP) and α-tubulin. The positions of the 116 kDa uncleaved PARP product and the 85 kDa cleaved fragment are indicated to the left. **(C)** Control 786-O cells and 786-O cells stably expressing wild-type VHLp19 (WT VHL) were grown to confluence and either left untreated or treated with ultraviolet (UV) light and lysed one day later. Western blots were performed for PARP and α-tubulin as in **(B)**.

Prior experiments had showed that VHL expression protects 786-O cells from UV-mediated apoptosis [16]. To see whether various mutant VHL proteins share this property, a panel of VHL mutants corresponding to all VHL disease subtypes [19] was stably expressed in 786-O cells (Figure 1A, top panel) (VHL type 1: del 114-178 and RC 161/2 QW; type 2A: Y98H; type 2B: R167W; type 2C: L188V). As seen previously, cells expressing mutant VHL that is associated with type 1 VHL disease, as well as control cells had high levels of HIF-2α (Figure 1A, middle panel), the only HIF-α isoform that is expressed in the 786-O cell line [4]. The cell lines were subjected to UV treatment and apoptosis was observed via western blotting for poly (ADP-ribose) polymerase (PARP), with presence of an 85 kDa cleaved PARP fragment indicating apoptosis (Figure 1B, top panel). The control and type 1 mutant VHL cell lines containing increased HIF-2α showed higher levels of the cleaved PARP. One type 2B VHL mutant, R167W, which had previously demonstrated slightly higher levels of HIF-2α than other type 2 mutants in previous assays [19], also showed slightly elevated levels of the cleaved PARP fragment. As a control, untreated 786-O cells showed no observable cleaved PARP (Figure 1C). Thus, in 786-O cells, UV-mediated apoptosis appears to be correlated to levels of HIF-2α.

**Figure 2:**
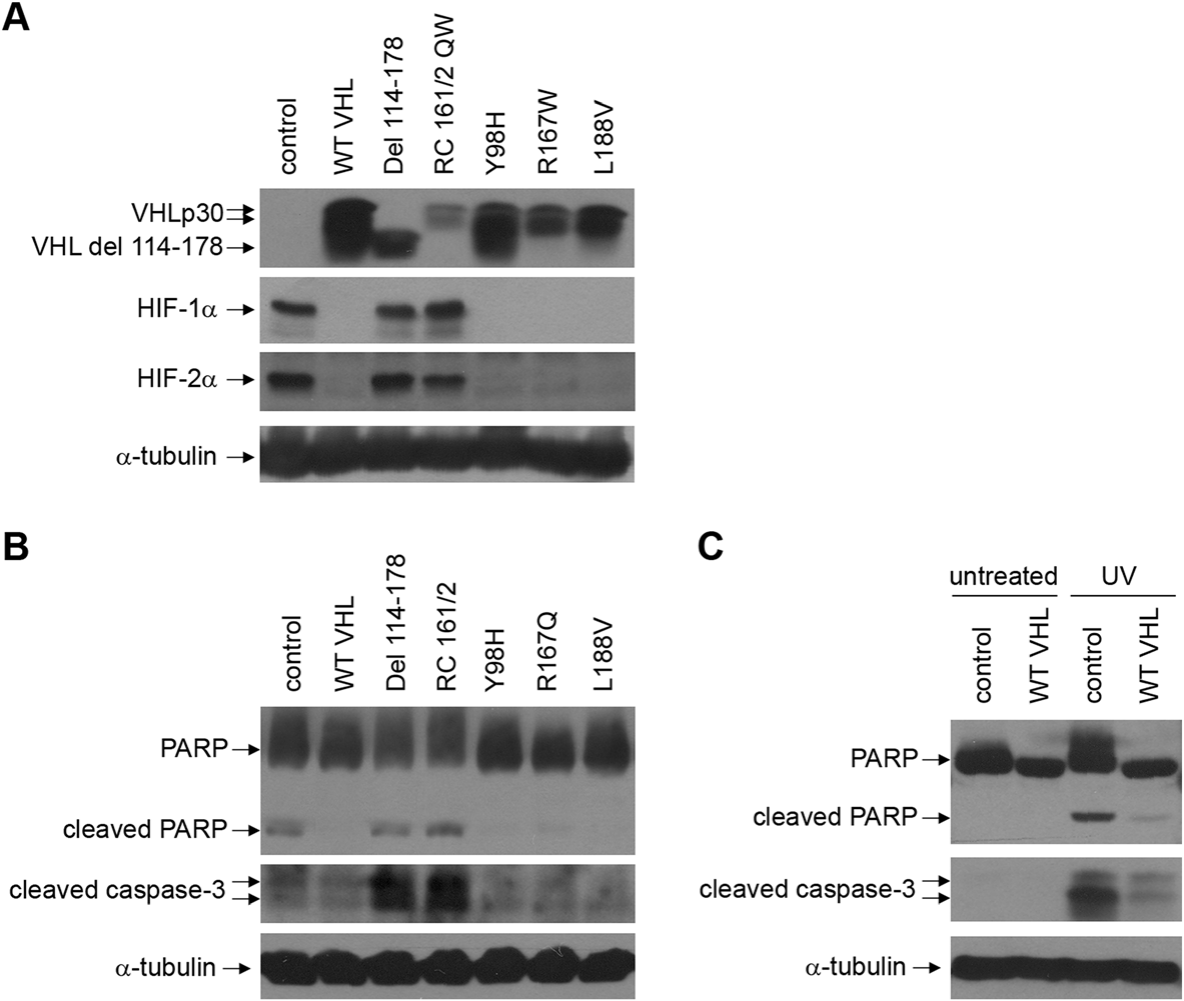
**Apoptosis in UV-treated RCC10 cells with mutant VHL proteins correlates to HIF-2α levels. (A)** RCC10 cells stably expressing either an empty vector construct (control), wild-type VHLp30 (WT VHL), or various mutant VHLp30 proteins [19] were grown to confluence and lysed. Cell lysates were equally loaded and separated by SDS-PAGE. Western blots were performed for VHL, HIF-2α, and α-tubulin. **(B)** The RCC10 cell lines in **(A)** were grown to confluence and treated with UV light and lysed one day later. Cell lysates were equally loaded and separated by SDS-PAGE. Western blots were performed for PARP, cleaved caspase-3, and α-tubulin. **(C)** Control RCC10 cells and RCC10 cells stably expressing wild-type VHLp30 (WT VHL) were grown to confluence and either left untreated or treated with ultraviolet (UV) light and lysed one day later. Western blots were performed for PARP, cleaved caspase-3, and α-tubulin as in **(B)**.

To determine whether this phenomenon was restricted to 786-O cells or more universal to renal carcinoma cells, RCC10 cells expressing the same constructs [19] were used (Figure 2A, top panel). RCC10 cells express both HIF-1α and HIF-2α [20] and control cells and cells containing VHL mutants associated with type 1 VHL disease showed tandemly up-regulated HIF-1α and HIF-2α (Figure 2A, middle panels). The cell lines were subjected to UV treatment and PARP western blotting (Figure 2B, top panel). Again, higher levels of PARP cleavage were seen in cells expressing type 1 VHL disease mutants. As a second assay for apoptosis, the cleavage of caspase-3, which occurs as cells execute an apoptotic program, was analyzed (Figure 2B, middle panel). The caspase-3 western blot was in agreement with the PARP blot, with noticeably more apoptosis in the type 1 VHL expressing cell lines that had increased HIF-α. As a control, untreated RCC10 cells showed no observable cleaved PARP or caspase-3 (Figure 2C). Thus, in both 786-O and RCC10 cells, UV-mediated apoptosis is greatest in cells with up-regulated HIF-α.

### Reduction of HIF-2α levels in 786-O provides partial protection from UV-mediated apoptosis

**Figure 3:**
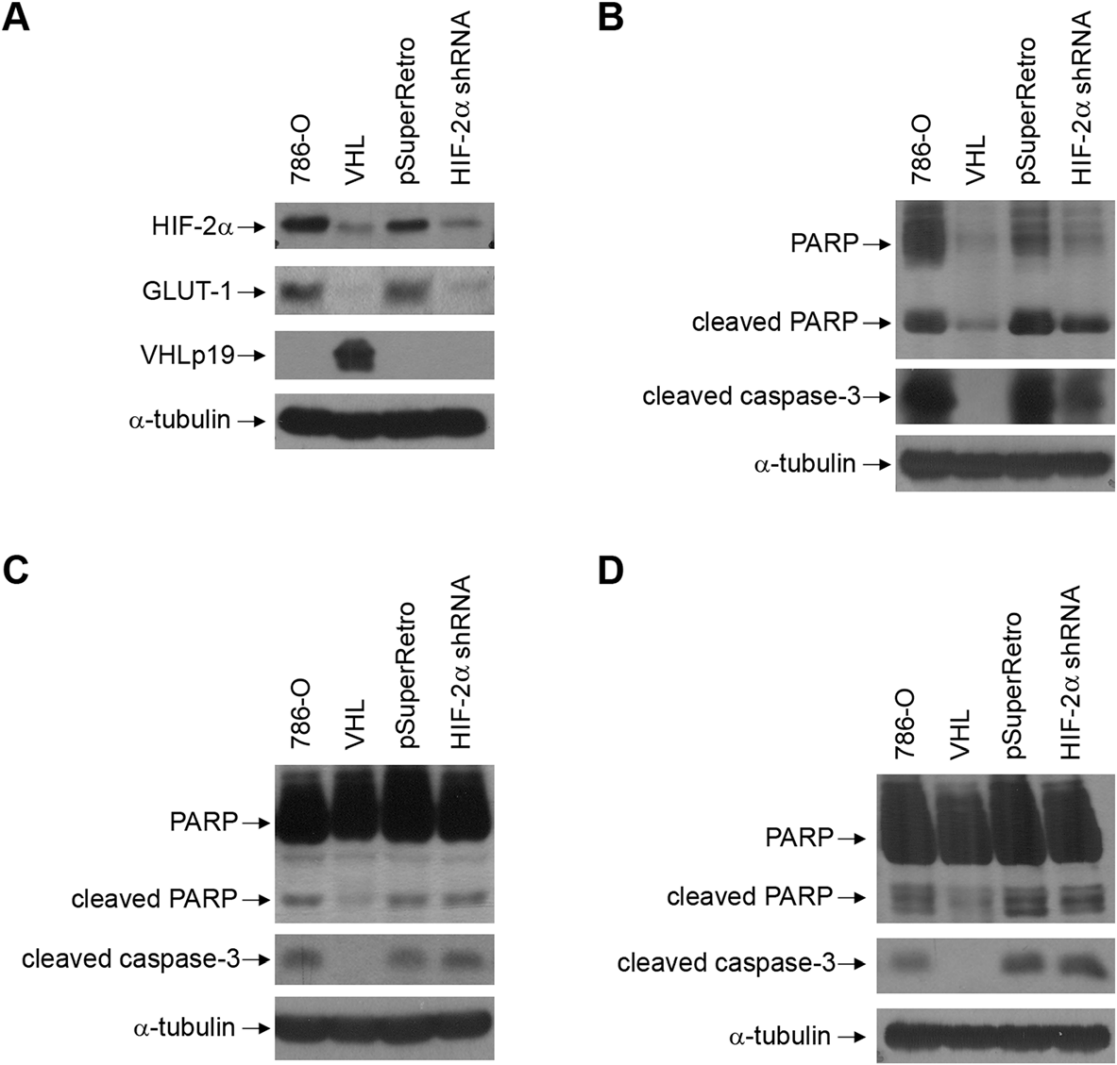
**Reduction of HIF-2α levels leads to protection in UV-triggered apoptosis, but not for apoptosis caused by glucose and serum starvation in 786-O cells. (A)** Parental 786-O or those either stably expressing wild-type VHLp19 or stably infected with a control vector (pSuperRetro) or a pool of two HIF-2α shRNAs vectors [21] were grown to confluence and lysed. Cell lysates were equally loaded and separated by SDS-PAGE. Western blots were performed for HIF-2α, GLUT-1, VHL, and α-tubulin. **(B)** The 786-O cell lines described in **(A)** were grown to confluence and treated with UV light and lysed one day later. Cell lysates were equally loaded and separated by SDS-PAGE. Western blots were performed for PARP, cleaved caspase-3, and α-tubulin. **(C)** The 786-O cell lines described in **(A)** were glucose starved for 24 hours as described in Methods. Cell lysates were equally loaded and separated by SDS-PAGE. Western blots were performed for PARP, cleaved caspase-3, and α-tubulin. **(D)** The 786-O cell lines described in **(A)** were serum starved for 2 days as described in Methods. Cell lysates were equally loaded and separated by SDS-PAGE. Western blots were performed for PARP, cleaved caspase-3, and α-tubulin.

To assay whether there is a direct contribution of HIF-α toward the apoptosis observed, a set of 786-O cells in which HIF-2α levels had been lowered by short hairpin RNA (shRNA) vectors [21] was employed (Figure 3). In cells containing the HIF-2α shRNA, protein levels of HIF-2α and the HIF-inducible gene product, GLUT-1, were comparable to the levels in cells in which VHL has been reintroduced (Figure 3A). UV treatment was performed on the cell lines. Compared to cells with a control vector, cells with lowered levels of HIF-2α had reduced abundance of cleaved PARP and cleaved caspase-3, although this was not decreased to the levels seen with cells in which VHL was replaced (Figure 3B). Thus, reduction in HIF-2α provided partial protection from UV-mediated apoptosis, indicating that its expression may either promote or be necessary for apoptosis after UV treatment.

To extend these analyses and determine whether the contribution of HIF-2α toward apoptosis was also observed with other forms of cellular stress, similar analyses were performed following conditions of glucose starvation (Figure 3C) and serum starvation (Figure 3D). Unlike with UV-treatment, reduction of HIF-2α levels had little effect on apoptosis caused by these stresses, whereas VHL was seen to exert a protective effect. Thus, in 786-O cells, VHL’s cell survival effects under glucose and serum starvation conditions are likely to occur independently of HIF-2α regulation.

### Reduction of HIF-1α and HIF-2α levels leads to differential effects on apoptosis in RCC10 cells

**Figure 4:**
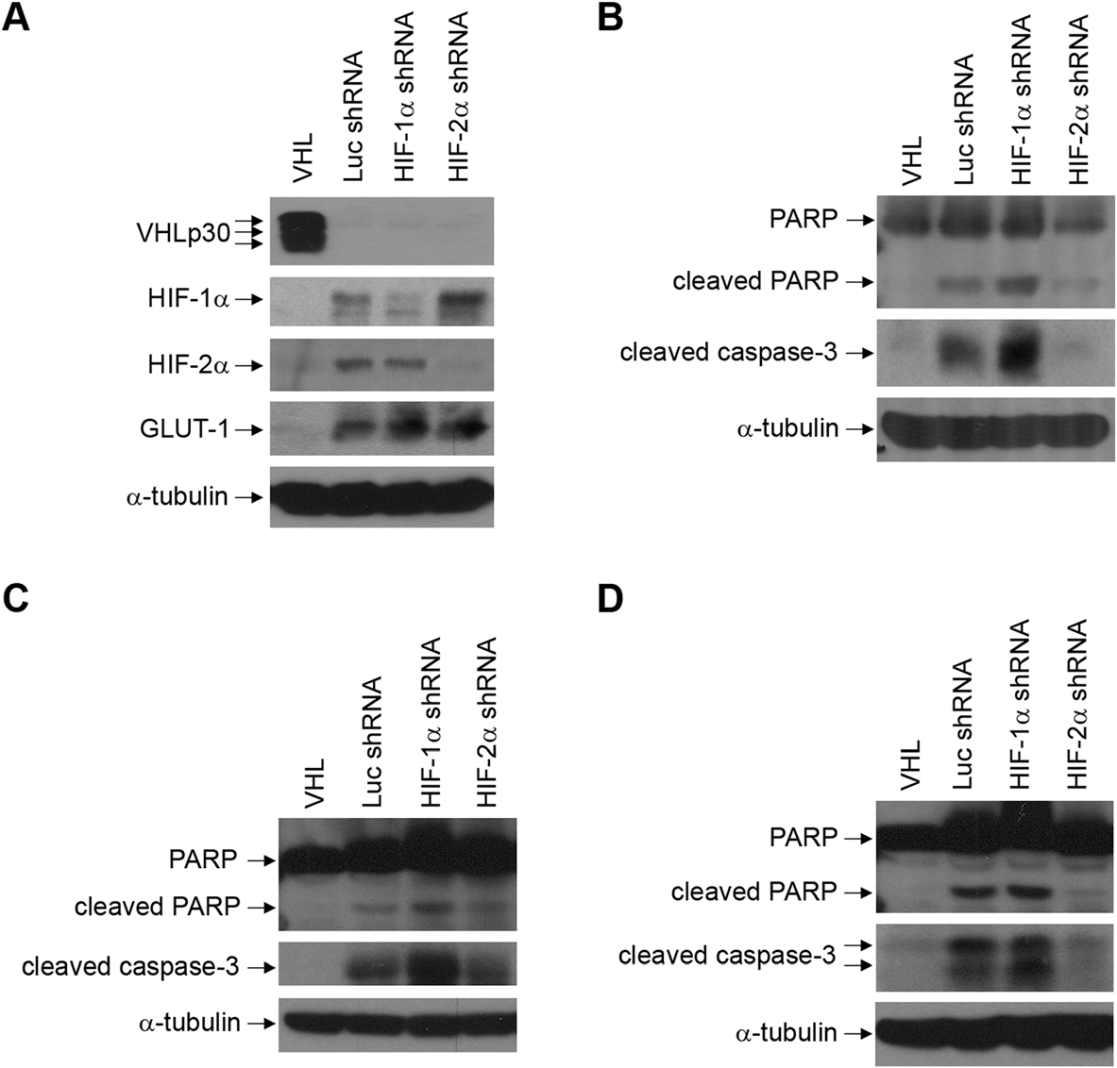
**Reduction of HIF-1α and HIF-2α levels leads to differential effects on apoptosis in RCC10 cells. (A)** RCC10 cells stably expressing wild-type VHLp30 or expressing a control shRNA targeting luciferase (Luc shRNA) or a pool of two shRNAs targeting either HIF-1α or HIF-2α were created. These cells were grown to confluence and lysed. Cell lysates were equally loaded and separated by SDS-PAGE. Western blots were performed for VHL, HIF-1α, HIF-2α, GLUT-1, and α-tubulin. **(B)** The RCC10 cell lines described in **(A)** were grown to confluence and treated with ultraviolet (UV) light and lysed one day later. Cell lysates were equally loaded and separated by SDS-PAGE. Western blots were performed for PARP, cleaved caspase-3, and α-tubulin. **(C)** The RCC10 cell lines described in **(A)** were glucose starved for 24 hours as described in Methods. Cell lysates were equally loaded and separated by SDS-PAGE. Western blots were performed for PARP, cleaved caspase-3, and α-tubulin. **(D)** The RCC10 cell lines described in **(A)** were serum starved for 2 days as described in Methods. Cell lysates were equally loaded and separated by SDS-PAGE. Western blots were performed for PARP, cleaved caspase-3, and α-tubulin. Similar results were seen with serum starvation for 4 and 6 days (data not shown).

Again, RCC10 cells were utilized to ascertain whether observations with 786-O cells were more generalizable among renal carcinoma cells. A set of stable RCC10 cell lines was created in which either VHLp30 was expressed or control, HIF-1α, or HIF-2α shRNAs were expressed (Figure 4). When VHL was expressed, HIF-1α and HIF-2α were noticeably down-regulated, as expected (Figure 4A, top 3 panels). Of note, in cells with lowered HIF-2α, HIF-1α levels were increased (Figure 4A, second panel). Knockdown of either HIF-1α or HIF-2α did not reduce levels of GLUT-1 (Figure 4A, fourth panel).

The RCC10 cell lines were subjected to UV treatment (Figure 4B). Similar to what was observed in 786-O, RCC10 cells with lowered levels of HIF-2α had decreased abundance of cleaved PARP and cleaved caspase-3, comparable to the levels seen in cells with reintroduced VHL. Interestingly, knockdown of HIF-1α caused an increase in cleaved PARP and caspase-3 as compared to control cells. Thus, in UV treated RCC10 cells, HIF-2α expression may promote apoptosis, whereas HIF-1α expression appears to inhibit it.

**Figure 5:**
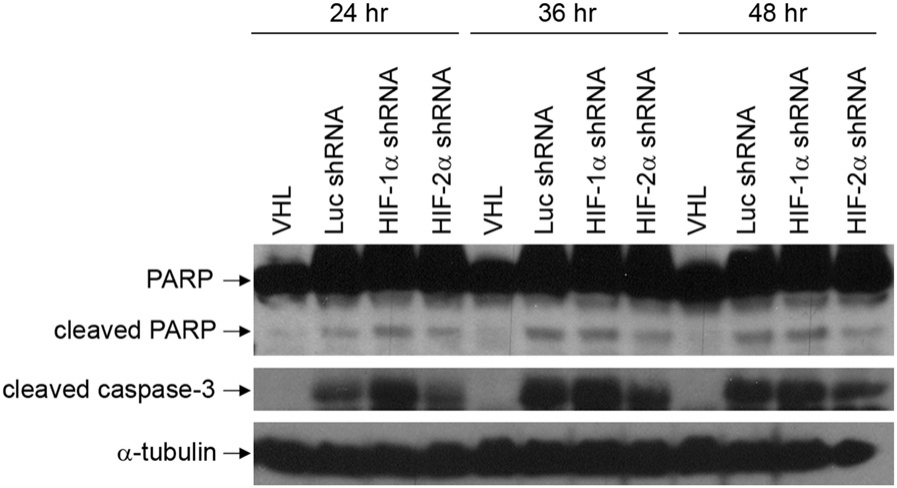
**Protection from apoptosis in cells with HIF-2α shRNA is seen for multiple glucose starvation time points.** RCC10 cells stably expressing wild-type VHLp30, shRNA targeting luciferase (Luc shRNA), or a pool of two shRNAs targeting either HIF-1α or HIF-2α were glucose starved for 24, 36, or 48 hours, as indicated above the blot. Cell lysates were equally loaded and separated by SDS-PAGE. Western blots were performed for PARP, cleaved caspase-3, and α-tubulin.

Apoptosis was also assayed for the RCC10 shRNA cell lines under conditions of glucose and serum starvation. Upon glucose deprivation, cells with lowered levels of HIF-2α showed decreased levels of cleaved PARP and caspase-3, whereas cells with lowered HIF-1α had increased levels of these apoptotic markers (Figure 4C). Protection from apoptosis in cells with HIF-2α shRNA was seen for multiple time points of incubation in glucose-free media (24, 36, and 48 hours), although this effect was slightly diminished with longer incubations (Figure 5). Under conditions of serum starvation, cells with lowered HIF-2α again had decreased levels of cleaved PARP and caspase-3 compared to control cells (Figure 4D). This effect was seen at multiple time points (2, 4, and 6 days in serum-free media, data not shown). Together, these results indicate that under conditions of glucose and serum starvation, removal of HIF-2α protects RCC10 cells against apoptosis, indicating that either HIF-2α expression promotes apoptosis or, given that cells with HIF-2α shRNA had higher HIF-1α levels, that HIF-1α expression may inhibit apoptosis caused by these cellular stresses.

## Discussion

In VHL-null cells expressing mutant VHL proteins, UV-mediated apoptosis was correlated with type I mutations that had high levels of HIF-α. The distinct contributions of HIF-1α versus HIF-2α toward apoptosis were then investigated using VHL-deficient renal carcinoma cell lines and manipulation of HIF-1α and HIF-2α expression. Reduction of HIF-2α levels led to decreased apoptosis with UV treatment. This result implies that the expression of HIF-2α is pro-apoptotic in UV treated cells. In UV-treated RCC10 cells, reduction of HIF-1α levels had the opposite effect, increasing apoptosis, suggesting that the expression of HIF-1α is normally anti-apoptotic. Therefore, there appears to be distinct effects of the two HIF-α isoforms on apoptosis in UV-treated cells. HIF-2α promotion of apoptosis may be a more important determinant than HIF-1α with UV treatment because RCC10 cells that have up-regulated levels of both HIF-1α and HIF-2α were susceptible to UV-initiated apoptosis (see Figure 2). Alternatively, it is possible that there is a greater abundance of HIF-2α than HIF-1α in RCC10 cells that is responsible for this effect, although this cannot be discerned using available antibodies to these isoforms.

The influences of HIF-1α and HIF-2α expression on apoptosis caused by additional stresses were also determined. For serum and glucose starved cells, knockdown of HIF-2α had little effect in 786-O cells, but decreased apoptosis in RCC10 cells. One important difference between these cell lines is that 786-O cells express only HIF-2α, whereas RCC10 cells express both HIF-α isoforms. Notably, knockdown of HIF-1α in RCC10 cells led to increased apoptosis in glucose-starved cells indicating that HIF-1α is anti-apoptotic for this stress. Moreover, there was an apparent increase of HIF-1α in RCC10 HIF-2α knockdown cells that coincided with reduced apoptosis. Thus, it is possible that the anti-apoptotic effects of HIF-1α are responsible for at least part of the reduction in apoptosis seen in HIF-2α shRNA cells upon glucose or serum withdrawal. This notion would also provide an explanation for the apparent lack of effect of HIF-2α shRNA in glucose and serum starved 786-O cells. However, regardless of the exact mechanism, these findings indicate overall differential influences on apoptosis by the HIF-α isoforms under serum and glucose starvation conditions, similar to UV irradiation.

While the same general trends were observed using 786-O and RCC10 cells, there were some slight differences in apoptosis (additional to the one mentioned above) that may relate to the HIF-α isoforms in these cells. For example, with 786-O cells, serum starvation caused the smallest induction of apoptosis out of the three stimuli used here, with longer exposures on western blots needed to visualize the cleaved apoptotic markers (data not shown). This effect was not observed with RCC10 cells. 786-O cells were in general more susceptible to apoptosis due to glucose starvation, with incubations past 24 hours resulting in cells that detached from the culture dishes, albeit less so for cells containing VHL (data not shown), whereas incubations of RCC10 cells in glucose-free media were possible for over 48 hours. While the lack of expression of the anti-apoptotic HIF-1α may be invoked for the latter finding, it is also likely that 786-O cells are more malignant than RCC10 (as seen by shorter doubling time for 786-O cells, data not shown), which may also result in a greater need for glucose and increased resistance to serum withdrawal. Of note, these observations also highlight the notion that different apoptotic regulators and/or pathways are likely to be engaged for the different apoptotic stresses. Note that although the apoptosis reported here may occur by different pathways, these pathways all converge on activation of caspases. Caspase-3, used as a marker in this report, is a central player in the apoptotic cascade, functioning as an effector caspase [22]. Cleavage of the other apoptosis marker used here, PARP, is downstream of caspase activation (reviewed in [23]), although PARP can also be cleaved by caspase-7 [24]. In the majority of assays here, PARP and caspase cleavage coincided, adding strength to their validity.

There are likely many factors that may account for the opposite effects of HIF-1α and HIF-2α on apoptosis observed in this study. Differences in the genes that HIF-1α and HIF-2α transactivate or repress are more likely to be involved. For example, HIF-1α, but not HIF-2α, promotes expression of glycolytic genes, which may cause cells either to be more efficient at glucose utilization or allow them to utilize other molecules as energy sources, resulting in decreased apoptosis in glucose starvation conditions, in agreement with the present findings. Other differential transcriptional targets involved in apoptosis include BNip3, which has been reported to be up-regulated by HIF-1α [8,25], cIAP, which may be up-regulated by HIF-2α [9], and ARC (Apoptosis Repressor with a Card Domain), which is up-regulated by HIF-1α [18], to name a few. In addition, some of the differential effects of HIF-1α and HIF-2α on apoptosis may be related to non-transcriptional effects, perhaps through differences in protein interactions. Opposite effects of HIF-1α and HIF-2α on mdm2 binding and p53 transcriptional activity have been noted (reviewed in [26]). Different roles of HIF-1α and HIF-2α in regulation of mTOR, which is involved in cellular survival decisions, have also been proposed (reviewed in [26]). Promotion of c-myc activity by HIF-2α and not HIF-1α [11], which can potentiate apoptosis in some circumstances, may also be a contributing factor and is in agreement with the promotion of apoptosis by HIF-2α observed in this report. Given the complexity of HIF-1α and HIF-2α biology, it is very likely the underlying causes for the differences in apoptosis as a result of HIF-1α or HIF-2α expression observed here are multifactorial. Further studies to explore these mechanisms are indicated, especially since HIF-1α and HIF-2α’s effects on apoptosis are likely to have implications toward clinical interventions that depend on apoptosis as their mode of action.

## Methods

### Cell lines and cell culture

293T and 786-O cells were obtained from the American Type Culture Collection. RCC10 renal carcinoma cells were generously provided by Miguel Esteban (Imperial College, London). All cells were grown in Dulbecco’s Modified Eagle’s Medium (DMEM) containing either 10% Serum Supreme (BioWhitaker) or 10% fetal calf serum (FCS). Media was supplemented with penicillin-streptomycin (100 U/ml and 100 μg/ml, respectively).

### Retroviral expression vectors, retroviral infection and cell lines

Wild-type and mutant VHLp30 retroviral expression constructs have been previously described [19]. Two retroviral vectors directing expression of shRNA targeting HIF-1α were created in pSuperRetro as described [27], using CTGATGACCAGCAACTTGA as one 19-mer HIF-1α target sequence and GCCACTTCGAAGTAGTGCT as another target sequence. Two retroviral shRNA vectors targeting HIF-2α, also pSuperRetro-based [7], were generously provided by Dr. William Kaelin (Dana Farber Cancer Center).

To produce retroviral supernatants, the recombinant retroviral vectors were co-transfected with the retroviral packing plasmid, pCL-Ampho [28], into 293T cells, as previously described [19,21]. To create stable pools of retrovirally-infected cells, cells were incubated overnight in a mixture (1:1) of retroviral supernatants and fresh medium that was supplemented with polybrene (10 μg/ml). The mixture was removed and replaced with fresh media the next day. For the HIF-1α and HIF-2α shRNA retroviruses, a pool of the two retroviruses for each shRNA target was used (with equal amounts of each retroviral supernatant). Three days following infection, the cells were replated and incubated for 10 to 14 days in media containing puromycin (0.5 μg/ml) to allow for proper selection.

786-O cells stably infected with control or HIF-2α shRNA retroviruses or stably transfected with VHLp19 have been described previously [19,21]. RCC10 cells stably infected with wild-type and mutant VHLp30 constructs have been described previously [19].

### Apoptotic cell stresses

Cell stresses were applied to cells grown at confluency. For UV treatment, media was aspirated from culture dishes, which were then placed under the germicidal UV lamp (UVC, 254 nm wavelength) in a tissue culture hood for 20 seconds (approximately 60 J/m^2^). Media was replaced immediately after UV exposure and the cells were incubated for 24 hours prior to lysis. For glucose and serum starvation, the media was removed from culture dishes and the cells were rinsed with phosphate buffered saline (PBS). The PBS was removed and cells were incubated with either glucose-free DMEM (Gibco/Life Technologies, Grand Island, NY) supplemented with 10% FCS (for glucose starvation) or DMEM lacking FCS (for serum starvation). Cells were grown for 24-96 hours (depending on cell type and treatment), after which they were lysed.

### Western blotting

Cells grown on 60-mm culture dishes until confluent were rinsed with PBS and then lysed by incubating with 150 μl of lysis buffer (50 mM HEPES (pH 7.6), 250 mM NaCl, 0.5% Nonidet P-40, 0.5% Triton X-100, 5 mM EDTA, 1 mM phenylmethylsulfonyl fluoride (PMSF), 1 mM Na_2_VO_3_ and 2 μg/ml each of aprotinin, bestatin, and leupeptin) at 4°C for 30 minutes. Lysed cells were scraped with a plastic scraper, resuspended by pipetting, collected, and were spun down in a refrigerated microcentrifuge for 15 minutes to remove all insoluble material. The supernatant was collected and a Bradford protein assay (Bio-Rad, Hercules, CA) was performed to determine protein concentrations. Equal amounts of protein for each well of a gel, ranging from 25-50 μg among the different blots performed, were mixed with an equivalent volume of 2× SDS buffer and were separated by SDS-PAGE. The separated proteins were then transferred to a polyvinylidene difluoride (PVDF) membrane overnight at 30 volts for 16 hours and western blotting was then performed as described.

### Antibodies

VHL mAb 11E12, which has been previously described [29], HIF-2α rabbit antibody (Novus Biological, Littleton, CO), GLUT-1 rabbit antibody (Alpha Diagnostic, San Antonio, TX), and caspase-3 and Bcl-2 rabbit antibodies (Cell Signaling, Danvers, MA) were used at a 1:1000 dilution in western blots. HIF-1α mouse antibody (BD Biosciences, Franklin Lakes, NJ) was used at a 1:250 dilution. PARP-1/2 rabbit antibody (Santa Cruz Biotechnology, Santa Cruz, CA) was used at a 1:200 dilution in western blots. Anti-mouse IgG-HRP and anti-rabbit IgG-HRP secondary antibodies (Southern Biotech, Birmingham AL) were used at a 1:2286 dilution.

## Conclusions

HIF-1α and HIF-2α exerted contrasting effects in all conditions tested. In UV-irradiated cells, HIF-2α expression promoted apoptosis, whereas HIF-1α was anti-apoptotic. In glucose or serum starved cells, HIF-1α expression was largely anti-apoptotic, while HIF-2α expression generally promoted apoptosis or had less of an influence, depending on the cell line used. These results reinforce that HIF-1α and HIF-2α have distinct biological roles. Moreover, their relative expression levels may influence some therapeutic interventions that are dependent on apoptosis and thus should be taken into account.
